# Some Gall-Bladder Cases Presenting Features of Interest

**Published:** 1912-03

**Authors:** E. H. E. Stack

**Affiliations:** Assistant-Surgeon, Bristol Royal Infirmary


### SOME GALL-BLADDER CASES PRESENTING FEATURES OF INTEREST.

BY

E. H. E. STACK, M.B., B.C., F.R.C.S.,

*Assistant-Surgeon, Bristol Royal Infirmary.*

DURING the last few years I have had some gall-bladder cases which have had peculiar features in diagnosis or treatment, and these may prove interesting.

**Case 1.**—A woman of 48 had a typical history of frequent attacks of colic, getting gradually worse, a little temperature occasionally, and sometimes slight jaundice, never deep or persistent. At the operation I found several stones in the common duct. The gall-bladder was buried in very dense adhesions, which took a long time to get through, and was very small and contracted with walls almost half an inch in thickness. It contained a small amount of brown inspissated mucus, but no stones. I removed it. In spite of the chronic condition which must have been going on for years, she had never been laid up by any of her attacks.

**Case 2.**—A woman, aged 51, had had mild attacks of pain for years. On operating I found the largest stone I have ever seen in the common duct. It was over 2 inches long, nearly 1 inch across, and weighed when dry 280 grains. In spite of its size, she had never had jaundice.

**Case 3.** —A woman of 56 had suffered for years with abdominal pain and indigestion, never an attack like colic. For three months before I saw her she had had jaundice, and was very thin, weak and ill. A lump could be felt in the gall-bladder region, and I thought she had cancer. She readily consented to an exploratory operation. After burrowing through dense adhesions, I suddenly found a gall-stone in the wound about the size of a small marble ; later on I found the gall-bladder and opened it. It contained no stones. I was quite unable to find any way out of it into the common duct, and on account of adhesions, could not demonstrate the duct from below. She was not in a condition to stand a very prolonged operation. For six weeks afterwards she had no sign of any return of bile in the stools, and was still discharging some from the wound. The morning she left the home there was for the first time a trace of colour in the fæces, and then she rapidly got better. Six months afterwards her doctor told me she was perfectly well. This case makes one think one should almost always give a case of deep jaundice the benefit of an exploratory operation.

**Case 4.**—A man of 34 had for many months attacks in every way typical of gall-stones. I expected to find numerous small calculi in his gall-bladder. The whole biliary apparatus, however, was perfectly normal ; but I found on the under surface of the liver a swelling, and on opening it saw it was packed full of hydatid daughter cysts. It was peeled out without much difficulty and very little bleeding. Subsequently I discovered that he had been in Australia seven years previously.

**Case 5.**—A woman, aged 37, for seventeen years had had constant slight attacks of colic, and on one occasion only had she had jaundice. She had seen many doctors in England and Germany, and opinions seem to have been divided as to whether her attacks were gastric or biliary. She was averse to operation, and left Clifton. Two years later she went to see a physician in London for a pain in her hip, and he discovered that she had a swelling in the region of her gall-bladder, which had never been present when I saw her. She came back to see me, and there was then quite a large swelling, feeling like a gall-bladder, and which was quite painless ; in fact, she had not had any of her usual pain for some months, and had begun to think she had got over the trouble. I found on operating an enormous gall-bladder, very thick, and containing over half a pint of pus and also about a dozen stones the size of marbles ; the ducts were all free. I removed the bladder, having to dissect it out of the liver, where it had got buried. Unfortunately I did not have a culture made. This was over a year ago, and she has had a few attacks of pain since like her old pain, but not enough to lay her up.

**Case 6.**—A man of 40, who for some years had had dull aching pain in the liver region, never definite colic or jaundice. The pain during the previous few months had become much worse, there was a slight persistent temperature, and resistance in the region of the pain could be felt. At the operation I found the most difficult mass of adhesions I have ever met with in this region. I simply could not define the gall-bladder, and presently opened it quite unexpectedly. It was normal in size and full of pus and stones. I then cut it away in pieces ; and when I got down to the neck it was better defined, and I could see that I cut across the cystic duct. In spite of the difficulty I had with the adhesions, I did not have to put a ligature on a single vessel, even when cutting off the neck of the bladder. The convalescence of this case was the most prolonged of any. He was laid up in bed for nearly six weeks, and then at home for a few more, before he was able to get back to his business.

**Case 7,** the last on my list, is perhaps the most interesting of all. She was a woman of 53, who had fair general health, except a little indigestion. Last September she had an attack supposed to be biliary colic, another similar one in November, and her third shortly before her admission to the Royal Infirmary on the 10th of January. Jaundice was then present, and had also been with her first attack. Tender resistance made it difficult to feel much in the upper abdomen, and her temperature was much raised. She was very ill and had a very feeble pulse. At the operation the gall-bladder was normal ; the fat in the wound area showed numerous spots of necrosis, such as one usually sees in acute pancreatitis. Adhesions to the left of the gall-bladder were separated, and a large foul abscess was opened which extended behind the stomach and back to the pancreas, which could be felt lying bare, but not in any way feeling abnormal. There was no evidence of duodenal or gastric ulcer or of stone in the common duct. A drain was put in, and later the cavity washed out daily. No attempt at healing took place, and the wound got into the sloughy condition common in pancreatic injury or ulceration. The wound was dressed with normal horse serum in the hope that this would satisfy the digestive action of the pancreatic juice and so save her own tissues. It is doubtful if this had much effect. She gradually got weaker, and died about four weeks after the operation. Examination showed the stomach quite normal, and the whole pancreas inflamed, ragged and suppurating.

All the other patients did well.

To sum up the points of interest :—

1. An advanced condition of cholecystitis, with dense adhesions or even pus in the gall-bladder, may be present in patients who have never had to leave off their ordinary duties.

2. Histories typical of gall-stone colic may be present in patients when there is nothing amiss with their biliary apparatus, as in the pancreatic and hydatid cyst cases.

3. A very large calculus can be present in the common duct without there ever having been jaundice.

4. In acute pancreatic disease, if the patient lives some time, the digestive action of the discharge in retarding healing is very marked.

5. Six weeks may elapse after an operation before bile begins again to enter the intestine.

6. Gall-stones can simulate cancer in all its symptoms, except, of course, those due to metastasis.

